# NeuTu: Software for Collaborative, Large-Scale, Segmentation-Based Connectome Reconstruction

**DOI:** 10.3389/fncir.2018.00101

**Published:** 2018-11-13

**Authors:** Ting Zhao, Donald J. Olbris, Yang Yu, Stephen M. Plaza

**Affiliations:** Janelia Research Campus, Howard Hughes Medical Institute, Ashburn, VA, United States

**Keywords:** NeuTu, connectome, electron microscopy, proofreading, segmentation

## Abstract

Reconstructing a connectome from an EM dataset often requires a large effort of proofreading automatically generated segmentations. While many tools exist to enable tracing or proofreading, recent advances in EM imaging and segmentation quality suggest new strategies and pose unique challenges for tool design to accelerate proofreading. Namely, we now have access to very large multi-TB EM datasets where (1) many segments are largely correct, (2) segments can be very large (several GigaVoxels), and where (3) several proofreaders and scientists are expected to collaborate simultaneously. In this paper, we introduce NeuTu as a solution to efficiently proofread large, high-quality segmentation in a collaborative setting. NeuTu is a client program of our high-performance, scalable image database called DVID so that it can easily be scaled up. Besides common features of typical proofreading software, NeuTu tames unprecedentedly large data with its distinguishing functions, including: (1) low-latency 3D visualization of large mutable segmentations; (2) interactive splitting of very large false merges with highly optimized semi-automatic segmentation; (3) intuitive user operations for investigating or marking interesting points in 3D visualization; (4) visualizing proofreading history of a segmentation; and (5) real-time collaborative proofreading with lock-based concurrency control. These unique features have allowed us to manage the workflow of proofreading a large dataset smoothly without dividing them into subsets as in other segmentation-based tools. Most importantly, NeuTu has enabled some of the largest connectome reconstructions as well as interesting discoveries in the fly brain.

## Introduction

Building the structural connectome of a brain is widely considered as an essential step of understanding the brain (Seung, 2012). Even if it is only a static snapshot of the brain without functional details, the information obtained from connectomes has been expected to provide unique and critical biological insights, as demonstrated in practice from the earliest efforts on *Caenorhabditis elegans* (White et al., 1986) to recent achievements on larger animals, such as *Drosophila melanogaster* (Takemura et al., 2013; Eichler et al., 2017), zebrafish (Wanner et al., 2016), and mice (Bock et al., 2011; Briggman et al., 2011; Lee et al., 2016; Morgan et al., 2016). Although the strategy of tracing neuronal skeletons has been widely used (Saalfeld et al., 2009; Boergens et al., 2017), segmentation-based reconstruction (Plaza et al., 2014; Kasthuri et al., 2015), or labeling every voxel in a volume, has its unique advantages. First, it is the most reliable way to get the complete reconstruction of a circuit, or at least it is easier to verify in dense reconstruction if some inconsistency is caused by reconstruction errors or biological randomness (Takemura et al., 2015). Second, it can leverage automated segmentation methods more easily to reduce manual work than skeleton-based sparse tracing can. Once segmentations are available, errors can be corrected by making local decisions without having to trace any long-range path of neuronal branches. For example, it is common that the border between two large segments to be merged is only a small fraction of the segments’ overall surface area. Confirming merges by examining small contact regions can often produce a high-quality long segment with much less effort than manual skeleton tracing. Third, it provides more detailed information about neuronal morphology, which cannot only facilitate quality control, but also play an important role in simulation (Hines and Carnevale, 1997).

The workflow of segmentation-based connectome reconstruction typically involves EM image acquisition, image pre-processing, automated segmentation, and manual proofreading. While each of these steps is technically challenging, the last step, manual proofreading, usually consumes the most human labor, which can become extremely expensive and time-consuming as the dataset is scaled up to the whole brain. Even though there are significant efforts of improving automated segmentation to reduce the work load of manual proofreading (Beier et al., 2017; Januszewski et al., 2018), manual proofreading is still currently the primary bottleneck. Improvement on manual proofreading is usually generally applicable and expected to save tremendous resources regardless of what automated segmentation algorithm is applied in the pipeline.

Due to the necessity of manual proofreading and its complexity, it is not surprising that various software tools, including Raveler (Olbris et al., 2018), Knossos^1^, Dojo/Mojo (Haehn et al., 2014), Eyewire^2^ and VAST (Berger et al., 2018), have been developed almost in parallel for correcting segmentation for dense or sparse reconstructions. While they have been successfully applied to produce local connectomes, recent advances in EM imaging (Briggman and Bock, 2012; Eberle et al., 2015; Hayworth et al., 2015; Xu et al., 2017) and segmentation (Beier et al., 2017; Januszewski et al., 2018) suggest new strategies and pose unique challenges for tool design to accelerate proofreading. For example, to the best of our knowledge, there is a lack of tools designed to operate on large segmented 3D objects freely without special constraints on the data, such as separating data into blocks (e.g., Raveler and Eyewire) or fixing errors slice by slice (e.g., Dojo and Mojo). Modifying, or mutating segmentation data in three dimensions is critical for providing a scalable solution for densely proofreading a large connectome.

Therefore, we have developed NeuTu to enable scalable proofreading on segmented datasets. Like many other proofreading tools, proofreading in NeuTu consists of a series of merges or splits. But unlike those tools, NeuTu has different approaches for scalable 3D object visualization and splitting, enabling intuitive operations in three dimensions. NeuTu has been used to both densely and sparsely proofread multiple regions of the fly brain, including connectomes of seven columns in medulla (Takemura et al., 2015) and the alpha lobe of the mushroom body (Takemura et al., 2017a).

## Materials and Methods

Table 1 provides terminology we used to describe our proofreading pipeline and software in this paper.

**Table 1:** Terminologies used in our proofreading workflow.

Terminology	Definition	Comment
DVID	A distributed, versioned, image-orientated dataservice developed by the Janelia Fly EM project team.	See https://github.com/janelia-flyem/dvid for more details.
3D image/volume	A function defined on a finite 3D grid: {1,…,*L*}×{1,…,*M*}×{1,…,*N*}→*I*	*I* is usually an integer value.
(Image) Block	A 3D image, when referred to as a subset of a larger 3D image.	
Grayscale image/data	The original image used for producing segmentation results.	In practice, they are registered and contrast-adjusted images acquired from electron microscopy.
Segment	A region labeled by segmentation to represent the same object, which is a neuron in our application.	A false merge means that a segment has voxels from different neurons. A false split means that voxels from multiple segments belong to the same neuron.
Body	A 3D segment.	
Sparse volume	A volume that has been compressed by ignoring background voxels.	
Multi-scale data	Data represented at different scales, in which a higher scale representation is a downsampled form of a lower scale representation.	A typical specification of scales is that the (n+1)*th* scale is downsampled by 2 from the n*th* scale.

### Collaborative Proofreading Workflow Based on Segmentation

**FIGURE 1:**
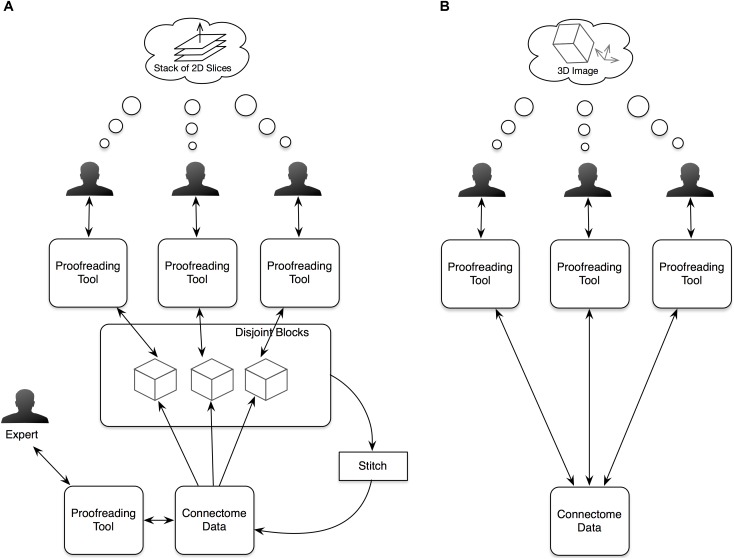
In our old proofreading workflow **(A)**, we partitioned image data into blocks and assigned the blocks to multiple proofreaders. The proofreading results from the blocks were then stitched and further proofread by an expert. The new workflow **(B)** simplifies the procedure by supporting simultaneous proofreading on the same dataset. Another significant improvement in the new workflow is providing visualizations and interactions in 3D space to help users view 3D segmented objects more naturally instead of as stacks of 2D slices.

NeuTu has been designed and improved continuously based on our current workflow of proofreading large-scale segmentation results. Before developing NeuTu, we used Raveler to proofread connectomes, such as the single-column medulla reconstruction (Takemura et al., 2013). Raveler was developed to handle a block-based workflow, in which the whole data are divided into disjoint blocks and different proofreaders worked on these blocks in isolation (Figure 1A). With the rapid increase of image size and improvement of automatic segmentation, which benefited from both advances in deep learning and innovative imaging technologies, however, it is difficult to manage the block-by-block workflow without a proportional increase of the overhead cost of dividing and reintegrating the data. One major difference resulting from these changes is that a segment can occupy many blocks. Fixing an error in a given block, especially a false merge error, becomes cumbersome when only a small portion of the segment is visible in the block. Many errors need a larger context to identify. Therefore, it is critical to visualize or manipulate a 3D segment or body with a global context.

For a segmentation-based workflow, the input is a set of segments, each composed of a set of voxels, and proofreading will output a new set of segments by reassigning voxels. Although this basic assumption remains the same, the major change in the current work is that the input segments are produced from 3D segmentation directly instead of a two-step process of 2D segmentation and linking. It implies that the typical way of fixing errors on individual planes followed by updating linkages is no longer a suitable option. Since we are also dealing with isotropic data, such as images acquired from focused ion beam scanning electron microscopy (Xu et al., 2017), there should be no predefined principal direction for a 3D segmentation. The old pipeline relied on 2D segmentation slices with a preferred planar direction for slicing because anisotropic data acquired from the widely used transmission electron microscopy often has the XY resolution one order of magnitude higher than the Z resolution, naturally leading to data management that uses the *Z*-axis as the principle direction to see image details more conveniently. A better workflow should be free of this constraint, encouraging human proofreaders to perceive a segment as a 3D object without worrying about its underlying representation.

More specifically, proofreading workflows that work on large datasets with high-quality segmentation requires the following functions, which NeuTu implements (Figure 1B):

(1) Allowing multiple users to proofread the same dataset in parallel without worrying about generating inconsistency;
(2) Efficient and high-quality 3D visualization of mutable segments, which are subject to modification at any time;
(3) Intuitive interaction with 3D segments;
(4) Ways of marking proofreading progresses of individual bodies as well as the overall connectome.

### Architecture of NeuTu

**FIGURE 2:**
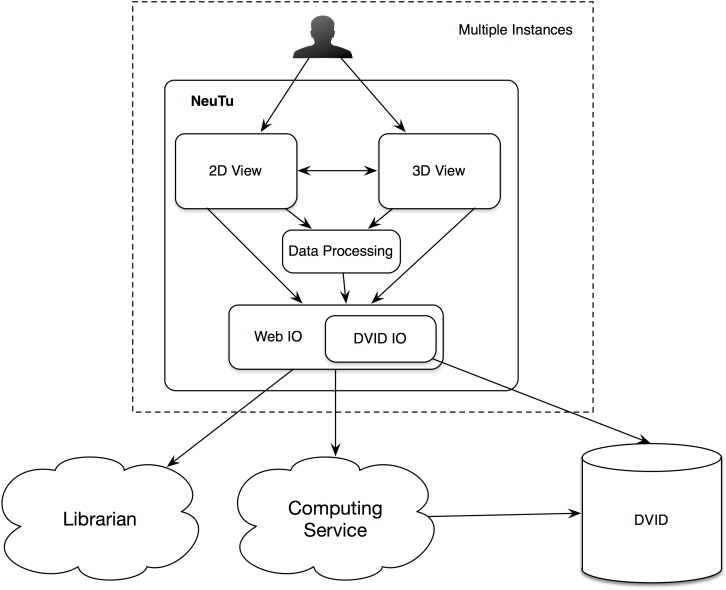
Architecture of NeuTu as a client of DVID and other remote services, include the Librarian for coordinating body assignment and the Computing Service for updating accessory results with data fetched from DVID.

Since NeuTu is supposed to be used on multiple computers at the same time for proofreading a shared dataset, designing it as a client of a data service is a natural choice. Specifically, we built it as a client of a distributed, versioned, image-oriented dataservice (DVID)^3^, which provides a fast IO access to large-scale 3D image data. Many functions in NeuTu are tuned to exploit important features of DVID, such as versioning, and optimized for data formats that DVID provides. On the other hand, NeuTu is a GUI application, which allows the user to interact with data as intuitively as possible. To this end, we provide two visualization modes in NeuTu. The 2D View provides a slice-based display of image and annotation data, while the 3D View shows those data in the 3D space. These two views can control each other through a direct communication channel to facilitate navigation. A command issued by the user will be passed from the front-end view to a lower-level IO directly or through a data processing engine. Changes in the data will be returned to update the views accordingly. Information related to the change, such as which bodies have been modified, may also be sent to the Computing Service for updating non-critical but useful results such as skeletons, which are usually expensive to compute. Although the DVID service can receives inputs from multiple users, it does not prevent users from interleaving merge or split results inconsistently. Therefore, we introduced a service called the Librarian to coordinate the workflow of multiple users. The Librarian allows a user to lock a body to keep it from being modified by other users, and unlock it when it is done. This lock-based coordinating workflow is well-suited for managing segments because each segment has a unique ID for the Librarian to track its status. Whenever a user wants to manipulate a body through NeuTu, the NeuTu client will ask the Librarian to lock the body first. If the body has already been locked by another user, the Librarian will return an error message, keeping the client from modifying the body. The overall architecture is illustrated in Figure 2.

### Feature Highlights

#### Data Management and Flow via DVID

All major data, including the original EM volume images (grayscale data) are stored in DVID. In this sense, NeuTu is a client of the DVID server. Since NeuTu fetches data from DVID on demand, the difficulty in handling a large connectome on the client side can be minimized. For example, to merge two bodies, NeuTu only needs to send DVID a request containing the IDs of the bodies, without having to deal with actual voxels. Body size is not a big issue for merging because the computation is as trivial as assigning a new ID to all voxels to merge, which is done by DVID and does not involve any inter-voxel relationship. In the case of visualizing or splitting a body, where big body size becomes a challenge for computation, we store the binary mask of each body separately as a sparse volume (i.e., only foreground voxels are recorded) in DVID. Each sparse volume is further compressed with run-length encoding (RLE). When a user wants to split a body, NeuTu can just download the binary mask and relevant grayscale data that contains EM signals. In DVID, images are stored as small fixed-sized blocks, with a typical size of 32 × 32 × 32 voxels or 64 × 64 × 64 voxels, for fast indexing. Retrieving a whole DVID block is usually faster than retrieving the same number of voxels distributed across multiple blocks. For optimal performance, NeuTu uses the same kind of block structure to manage grayscale data for a body. When a block contains voxels both inside and outside of the body, all the grayscale data in that block will be retrieved and stored in memory for further usage. Conforming to the block alignment by using a little more memory space to retrieve and store block-aligned data can lead to speedups since unnecessary data slicing is avoided in DVID.

NeuTu takes advantage of the sparse volume representation provided by DVID to allow manipulation of individual bodies. For example, when a body is selected, NeuTu downloads the binary mask of the body with RLE, which is typically much smaller than a list of individual voxel locations, allowing it to fit in memory. The downloaded body data can be used in two ways that are critical for 3D body manipulation. First, surface points can be extracted from the body data and converted into a convenient form for 3D visualization. The user can select any of the surface points in 3D to perform a further operation such as exploring grayscale data at the corresponding position or adding a bookmark at the position directly. Second, the sparse body data can be used as a mask for constraining the range of watershed-based split computation, thus reducing computational time significantly. Although the final watershed step has to be run over the bounding box around the body for computational efficiency, some pre- or post-processing steps such as downsampling or connected component analysis can be applied to the sparse form directly.

#### 3D Body Representation and Visualization

3D visualization is the most critical component of NeuTu to achieve the goal of intuitive operation in 3D. Showing a neuron in 3D allows the user to examine the reconstruction much more easily than displaying a slice. The user can click a surface point on the reconstruction and jump to that location in the 2D view. This is very helpful for examining problematic branches. For example, in some dataset, a branch terminal without synapses often means that a part of the branch is missing. The user can easily see the point and perform more careful examination in the 2D view.

**FIGURE 3:**
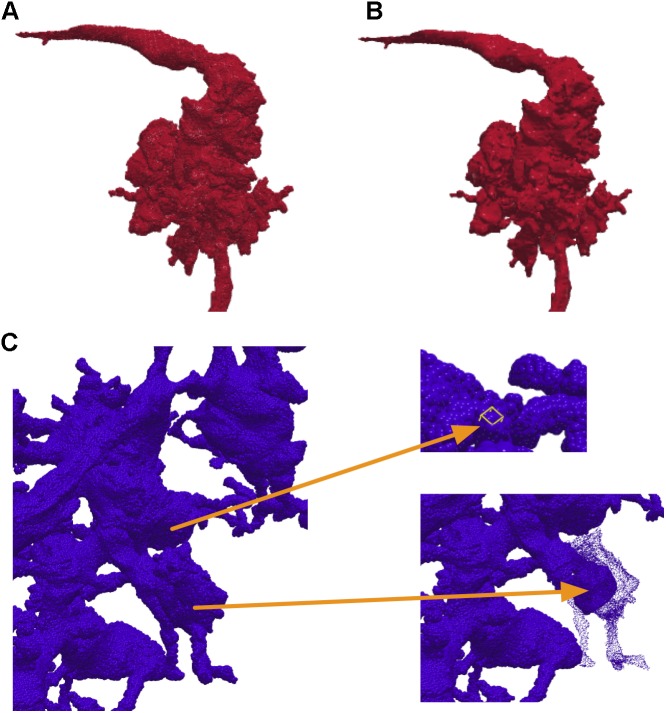
NeuTu can render a body with a collection of surface spheres **(A)**, which has overall shading similar to mesh rendering **(B)**. Any of the spheres can be selected for further operation such as localization or size adjustment **(C)**.

In NeuTu, besides mesh visualization, 3D visualization of a body is also implemented by rendering 3D surface points of the body as a set of spheres with independent shading. Compared to other techniques such as mesh or volume rendering, rendering surface spheres has advantages in updating speed and intuitive interaction. Without the need to create faces and compute normals, surface points can be extracted more quickly than generating a mesh, so that loading a body into the 3D visualization engine takes shorter time when pre-computation is not an option. Overlapping spheres (Figure 3A) actually emulates surface shading (Figure 3B) naturally, thus saving the time of computing normals. Furthermore, the sphere representation provides an easy and intuitive interface for the user to select a surface point in 3D, which only needs a click on the corresponding sphere. Once a position is selected, the user can then quickly navigate to grayscale image nearby or add a bookmark directly at that position. The user can even select a collection of spheres and adjust their sizes to highlight some morphological features in dense arborization (Figure 3C).

**FIGURE 4:**
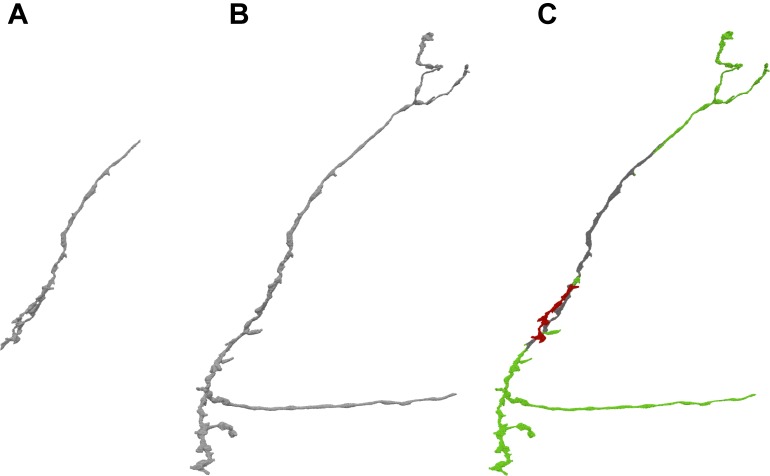
By comparing different versions of a given body stored in DVID, NeuTu is able to show proofreading history of the body with color coded parts. The figure shows the difference **(C)** between the current version **(B)** of the neuron and an earlier version **(A)**. Green sections in panel **(C)** show additions from the earlier version, red sections show subtractions, and gray sections are unchanged.

Instead of using pre-computed visualization primitives, NeuTu computes them to ensure consistency between the scene and its underlying data, which is subject to modification at any time. This requires fast computation of visualization data to reduce waiting time. Even though approximating with surface spheres helps, transferring and parsing data from the server can still be time-consuming for a large body. Therefore, we exploit the multi-scale data representation in DVID to allow a multi-scale updating strategy in NeuTu. When a body is selected for 3D visualization, its RLE data is fetched first from the lowest resolution representation, and then to the next higher resolution, until a certain size threshold is reached. Thanks to flexible version control in DVID, which allows us to set any checkpoint of proofreading results and create a new version from it, NeuTu is able to visualize body differences from different segmentation versions (Figure 4), which is particularly useful for tracking proofreading progress and for training.

#### Fast Interactive Segmentation

**FIGURE 5:**
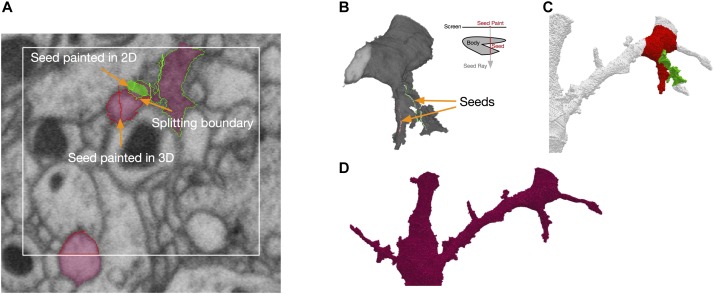
Splitting in NeuTu is powered by seeded watershed, which takes seeds painted by user in 2D **(A)** or 3D **(B)** as input. Note that painting a single point in the 3D view can generate multiple seed points by ray shooting **(B)**. Seed points outside of the body are ignored by split computing. Splitting results can be previewed in 3D **(C)**. The user can also paint a bounding box to accelerate computation **(A)**. NeuTu runs connected component analysis to assemble regions outside of the box and produce the final result **(D)**.

NeuTu uses a seeded approach for fixing false merges interactively. In this approach, the user needs to paint seeds on regions belonging to different neurons with different colors. The seeds can be painted in either the 2D view (Figure 5A) or the 3D view (Figure 5B). In the 2D view, a seed can be painted on any slice, and in the 3D view, a seed can be painted as a sequence of rays, each going through the target body from the first surface point to the last surface point it encountered on its path. The splitting results can be viewed in both views as well (Figures 5A,C).

To improve the speed of fixing false merges, we designed an efficient seeded splitting system on the client side. The system is powered by a highly optimized implementation of the seeded watershed algorithm. According to our benchmark test, our seeded watershed implementation is about as twice fast as that in Insight Segmentation and Registration Toolkit (ITK)^4^, a widely used image processing library. Because membrane voxels are generally darker than cytoplasm voxels, the watershed computation is applied on grayscale data directly by assuming that lower intensity has a higher “water level.” Although this may not be as accurate as using boundary maps or affinity graphs, especially when there are dark organelles near the boundary, saving computational overhead of edge enhancement leads to a good accuracy-speed trade-off in practice.

To further reduce computational time significantly for splitting a sparse volume, watershed computation is constrained to the body foreground. However, even excluding background voxels is often not enough for a quick turn-around. Therefore, NeuTu provides options of adding further constraints. The user can quickly check splitting results locally by trigging a local computation that only covers an area around the seeds. This provides a fast feedback for the user to adjust seeds accordingly, making less accurate segmentation more tolerable by making it easier to correct. Even though correct local splits do not guarantee correct global splits, it is a reasonable indicator of seed quality. Alternatively, the user can explicitly define a bounding area for splitting by painting a rectangle (Figure 5A). NeuTu can produce correct results if the bounding box contains the whole merging border, which is often constrained in a region much smaller than the bounding box of the body. Any piece outside of the local box will be attached back to the local split regions after connected component analysis (Figure 5D). Although it is not common, there may be multiple false merging spots that are far away from each other. The user can decide to split the segment progressively in this case.

It is possible that a body can have multiple disconnected components. Because watershed never crosses from one component to another, splitting such a body is the same as running watershed on each component independently and then joining regions that have the same watershed labels. If a component has no seed point on it, it stays with the origin body.

#### Synapse Editing

**FIGURE 6:**
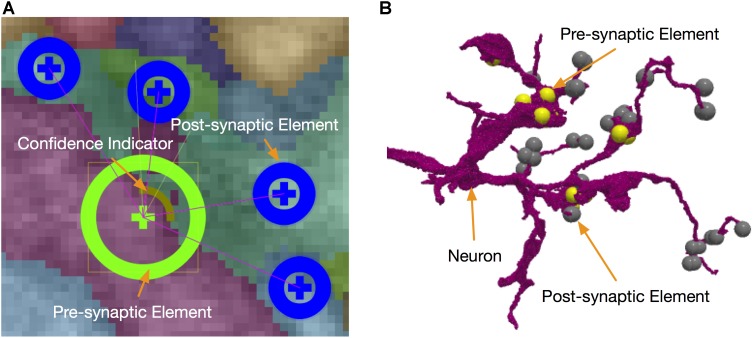
Synapses can be edited in either 2D **(A)** or 3D **(B)** with the assistance of informative visualization.

Besides neuron segmentation, synapse identification is also essential for building a connectome. Each synapse has a pre-synaptic element and a post-synaptic element to define a directed connection. In our system, synapses are stored in DVID as a kind of annotation data that can be queried by their coordinates. NeuTu reads synapses from DVID and displays them in both 2D (Figure 6A) and 3D (Figure 6B). The user can add a synaptic element at any position, connect/disconnect an element from/to another, or remove/move an element. Since synapse editing starts from automatic predictions, NeuTu adds some special visualization hints to the glyph of a synapse to indicate its confidence level or verification status.

#### Data Annotation for Workflow Management

Like any large-scale workflow, the workflow of large-scale proofreading should be organized to avoid duplicated work or blind spots. NeuTu provides various data annotation tools for such a purpose. In NeuTu, the user can annotate a body by giving it a biologically meaningful name and/or specifying its status. For example, the user can annotate a body with one of the seven pre-defined statuses, including “not examined,” “traced,” “traced in ROI,” “partially traced,” “orphan,” “hard to trace,” and “finalized.” We can assign bodies to different proofreaders according to the statuses. For example, if a body is annotated as “hard to trace,” we can assign it to an expert for further examination. The assignment is often done as a separate process outside of NeuTu. To help examine the bodies sequentially or as a group, NeuTu provides a table widget called the “sequencer” in which bodies can be filtered by regular expression or sorted by their properties such as synapse counts.

**FIGURE 7:**
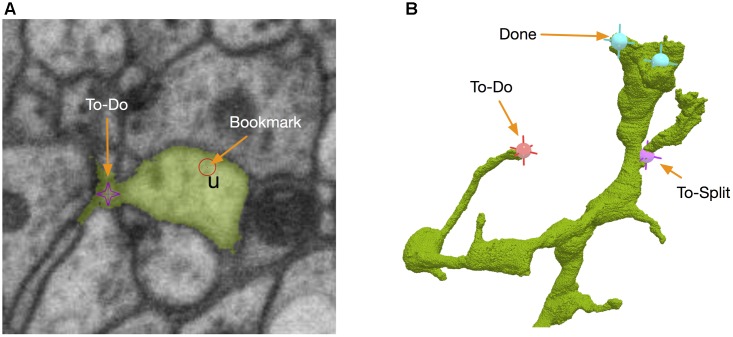
NeuTu uses point-based markers to facilitate proofreading workflow. For example, bookmarks are point annotations for labeling interesting locations **(A)**, which can be a place assigned for a double-check. To-dos are body-associated flags for tracking the proofreading status of a body. They can be edited in either 2D **(A)** or 3D **(B)**.

Those body-specific annotations do not tell where the problematic sites are. Therefore, we added another kind of annotations called bookmarks (Figure 7A). A bookmark is defined as a 3D point with a type and a comment. For example, the user can add a bookmark with a type “false merge” to specify that the corresponding location has a potential false merge error. A comment can be any text with more detailed information about the site. While bookmarks are handy for tagging interesting locations, they are only visible to their owners and are not necessarily used in proofreading. A special kind of location-specific annotation called reviewing marks is designed to show potential spots to proofread. Visible to all the proofreaders, those annotations have two statuses, to-do or done. To-do can be further labeled as “to merge” or “to split.” They serve as a check list in the proofreading workflow. When a user displays a body in the 3D window, he/she is able to see all the reviewing marks in the 3D visualization (Figure 7B), with different colors to distinguish to-do or done statuses. The user can add, delete or modify an annotation in 3D directly. The synchronization between annotations and body IDs is managed by DVID. Whenever a body ID is changed by merging or splitting, all annotations associated with the old ID will be updated automatically to use the new ID.

### Implementation

NeuTu is mainly written in C++, initially built upon the visualization and interaction engines from neuTube, software for tracing neurons in light microscope images (Feng et al., 2015). Important development updates introduced in NeuTu include replacing Qt4 with Qt5, allowing C++11 syntax to take advantage of modern C++ features, as well as using Conda Package Management^5^ for cross-platform deployment. The code is publically available on Github^6^.

## Results

**FIGURE 8:**
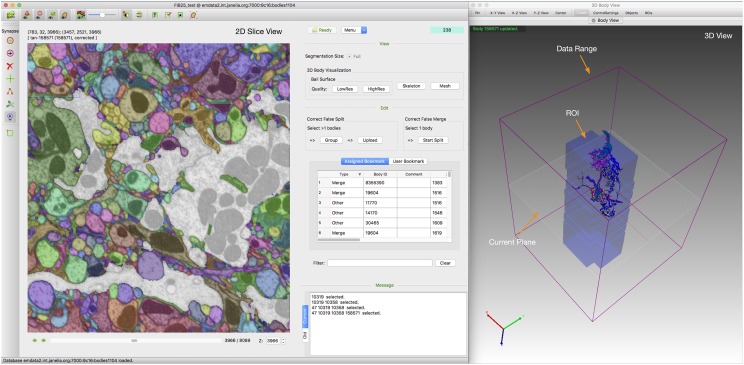
Overall GUI of NeuTu on Mac OS X with 2D and 3D windows side by side to enable efficient proofreading by showing global information as well local details. For more details, please refer to the user manual (see text footnote 8).

Designed to be cross platform, NeuTu has been built and tested on several modern Linux systems (Fedora 16+, Scientific Linux 7) and Mac OS X (10.12.6+). Figure 8 shows a typical GUI of NeuTu on Mac OS X. The user can interact with 2D and 3D windows side by side, assisted by glyphs in the 3D view to infer relative positions of selected bodies and the field of view in 2D. As shown in Table 2, our focus on scalable segmentation-based reconstruction and feedback from in-house proofreaders have led to a combination of unique features in NeuTu compared to other available proofreading software. More details about NeuTu functions can be found in our online user manuals, including a short manual for quick start ^7^ and a long manual for full details ^8^.

**Table 2:** Feature comparison for segmentation-based proofreading tools shows that NeuTu has a unique combination of features tuned to proofread large-scale dense connectomes.

Software	Data store	3D interaction^1^	Fixing false merge	Synapse editing	Real time collaboration
NeuTu	Server	High	3D seeded splitting	Synaptic sites and links	✓
Raveler	Local	Low	Supervoxel splitting/3D seeded splitting (limited^2^)	Synaptic sites and links	N/A
Knossos	Server	Low	N/A	N/A	N/A
Dojo	Server	Medium	2D splitting	N/A	✓
Mojo	Local	Medium	2D splitting	N/A	N/A
Eyewire	Server	Medium	N/A	N/A	N/A
VAST	Local/Server	Medium	2D splitting	Synaptic sites	N/A

*^1^High: 3D visualization, bookmark, proofread directly in 3D, localization; Medium: 3D visualization, localization; Low: 3D visualization. ^2^Slow computation, unable to handle large body.*

**FIGURE 9:**
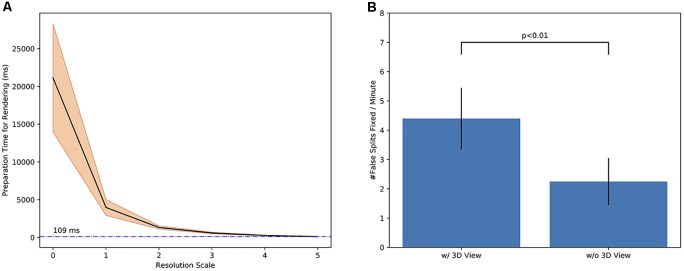
Experimental results showed that particular designs and strategies in NeuTu could help improve user experience and proofreading efficiency: **(A)** the waiting time for converting a body in DVID to geometric primitives for 3D rendering decreases exponentially as the downsampling scale increases, leading to real-time response (109 ± 66 ms) at scale 5 (downsampled by 32 along each dimension); **(B)** using 3D visualization can accelerate proofreading significantly, such as doubling the productivity in fixing false splits.

We evaluated how particular designs and strategies in NeuTu could help improve user experience and proofreading efficiency. The testing results obtained from 27 bodies showed that the multi-scale updating strategy described in Section “3D Body Representation and Visualization” could greatly improve response time (Figure 9A), which is measured by how long it takes to convert a body in DVID into geometric primitives for 3D rendering. Sampled from our 7-column medulla dataset (Takemura et al., 2015), the 27 bodies have 59 × 10^6^ voxels on average, with a range from 15 × 10^6^ to 16 × 10^7^ voxels. A typical body among them can be displayed in real time (∼100 ms latency) at the lowest resolution, which is good enough to show the overall shape. To test if 3D visualization is important for proofreading, we chose 10 incomplete bodies from a superset of the 7-column medulla dataset (Shinomiya et al., unpublished) and asked 10 proofreaders to trace from each body with or without 3D visualization within 2 min. Bodies were assigned randomly to each proofreader without duplication. The results showed that 3D visualization could almost double the efficiency of locating false splits by finding 4.40 ± 1.05 false spits per minute, compared to 2.25 ± 0.80 false splits per minute (*p* < 0.01) without the help of 3D visualization (Figure 9B). Note that 2D and 3D visualizations are more complimentary than exclusive. In some cases, such as in a severely over-segmented region, examining the 2D view may lead to more corrections than using 3D visualization only. 3D visualization becomes more heavily used when false splits are more sparsely distributed because of its advantage of showing global morphology. For example, one important feature to identify a false split is a branch terminal without synapses, which is difficult to miss in 3D visualization.

We have applied NeuTu to aid in the reconstruction of two EM datasets, the 7-column medulla dataset and the MB dataset, acquired from an optical lobe and a mushroom body of the fly brain, respectively. Table 3 summarizes the data and reconstruction results. More details of data acquisition and processing methods can be found in Takemura et al. (2015, 2017a). Most of the work for the 7-column medulla dataset was done in Raveler, while NeuTu was still under development. The initial connectome was later refined by sparsely tracing more than one hundred neurons in NeuTu, which has significantly better 3D visualization for finding false merges efficiently. The extra tracing made the neurons become more complete and reliable for biological analysis, allowing us to add seven new neurons to the NeuroMorpho database^9^ along with 525 neurons proofread by Raveler previously.

**Table 3:** Summary of published connectomes proofread by NeuTu.

Region	EM Volume Size (μm^3^)	Resolution (nm^3^)	Size of segmented region (μm^3^)	Skeleton length (mm)	#Neurons	#Synapses
Medulla	40 × 40 × 80	10 × 10 × 10	∼30 × 10^3^	∼278	1149^1^	∼53,500 presynaptic; ∼315500 postsynaptic
Mushroom body	180 × 180 × 480	8 × 8 × 8	∼176 × 10^3^	∼256	983	89,406 presynaptic; 224,697 postsynaptic

*^1^The number of neurons for the 7-column dataset is more than we reported in Takemura et al. (2015) because unidentified neuronal segments are also included here.*

For the MB dataset, automatically computed segments and synapses were initially proofread in Raveler, and then imported into DVID. A second round of proofreading, which involved real-time collaborative work of multiple proofreaders, was performed in NeuTu to create the final connectome. We used focused proofreading (Plaza, 2016) available in Raveler to proofread 903,309 potential false splits and then used NeuTu to trace 24,480 bodies potentially with false splits that were often trickier to identify automatically than manually. NeuTu was also used to correct false merges in 9,870 bodies, which was challenging for Raveler. The scale of either connectome is significantly larger than the one previously produced by Raveler alone, which has skeleton length amount to about 105 mm and 8637 synapses (Takemura et al., 2013). The number of synapses in each connectome is also at least one order of magnitude bigger than any other connectome that has been published, such as 4,657 synapses in Wanner et al. (2016) and 1,700 synapses in Kasthuri et al. (2015).

## Discussion

We have developed NeuTu for addressing emerging demands for connectome proofreading, such as managing big data smoothly, leveraging high-quality segmentations, and simultaneous collaborative proofreading. Our software has specific functions tailored to these needs. For example, the highly interactive 3D visualization provided by NeuTu allows the user to trace a neuron quickly with terminal examination. NeuTu has helped two large connectome reconstructions for deciphering vision and memory, respectively, in the fly brain. Both of them are one order of magnitude more complex than any other connectome in the literature in terms of the number of connections. In the connectome involved in visual processing (Takemura et al., 2015), accurate neuronal morphologies of the T4 neurons traced in NeuTu has revealed more details of the motion-detection circuit (Takemura et al., 2017b) and led to accurate prediction of motion-detection cells in *in silico* simulation (Gruntman et al., 2018). The other circuit, which is located in the memory center of the fly brain, is the most detailed connectome related to memory and learning to date. From that highly detailed connectome, we have not only confirmed the surprisingly random connections in sense information encoding, but also found some new connections that had never been observed before (Takemura et al., 2017a).

NeuTu is mature enough to be employed as a research proofreading tool without any further development, assuming automatic segmentation is available along with its registered EM data. However, some improvements may provide better user-friendliness and efficiency. One major limitation of NeuTu is that it is bound to DVID. This means that relevant data need to be imported into DVID first for NeuTu to work. Currently there is no easy plug-and-play interface for a user to proofread their data that are commonly stored as image files or in their own database format. This problem can be alleviated by providing a single script for converting common data formats into a DVID repository. Likewise, exporting proofreading data from DVID back to the format preferred by the user will also be useful. A more fundamental solution regarding this issue is to add an abstract layer to separate NeuTu from the actual APIs of a specific database. With such a layer, adding support of a new database to NeuTu would not require changing NeuTu itself. Such flexibility would help NeuTu scale too. For example, the number of users working on the same dataset is limited by the capability of DVID in our practice. Choosing more performance backends would be a reasonable option to match specific application goals that need to go beyond these limits.

Regarding performance on the client side, one challenge for NeuTu, or any other similar proofreading software, is efficient processing of big bodies (>100 M voxels). Even though we have employed strategies such as sparse volume representation, bounded splitting, and multi-scale updating to enable NeuTu to manipulate big bodies smoothly in most cases, there are some bottlenecks like data fetching and flood filling that are proportional to the body size. In practice, we used downsampling to limit the bounding box of a body to 1G voxels for responsive visualization and splitting. The major side effect of downsampling is the loss of morphological details, which can be addressed by using hybrid resolution scales for the same body. While we have not encountered any memory issue for common operations such as splitting or merging, which usually involves only a small number of big bodies, some unusual operations such as loading many big bodies (say, hundreds) into 3D visualization can indeed cause the machine to run out of memory. Potential solutions to this problem include optimizing related data structures, limiting memory usage, and automatically suggesting the user to visualize skeletons instead.

Future work could involve adding more functions for biological analysis, such as querying neurons and local circuits freely and allowing the annotation of more biological details (e.g., synapse sizes and subcellular structures). These features would be particularly useful at the late stage of proofreading, when the focus is shifting from an image-guided process to extracting biologically relevant circuits.

Being more intelligent is another important direction of NeuTu development. This will involve significant research work, like making suggestions based on global shape priors, as well as some simpler tricks, such as presenting nearby orphans segments automatically. But for most of those intelligent strategies, one common challenge will be harnessing fast computation to create a pleasant user experience. Tackling the challenge by optimizing code or algorithms for real-time computation is not generally practical because data involved are usually big. Extensive pre-computation will be necessary. Pre-computed results should be relatively light so that they can be uploaded into the database quickly without taking too much bandwidth and space. The results can be organized at different levels, such as skeletons at a lower level and skeleton similarity at a higher level, and do not need to be up to date. The NeuTu client should leverage such information, which can be noisy but has meaningful statistical patterns, to generate useful hints for the user.

Developing a proofreading tool does not only involve software development, it is also about designing proofreading strategies and workflows. Interestingly, this synergy between software design and reconstruction goals resulted in NeuTu software that can be used beyond the initial target application. For example, the flexible annotation system in NeuTu is directly shaped by the need of organizing collaborative proofreading, even though our main goal is to correct segmentation errors.

## Author Contributions

TZ and SP designed the project and wrote the paper with inputs from other authors. TZ led the software development and wrote most of the code. DO and YY developed part of software.

## Conflict of Interest Statement

The authors declare that the research was conducted in the absence of any commercial or financial relationships that could be construed as a potential conflict of interest.

## Footnotes

1 https://knossostool.org

2 http://eyewire.org

3 https://github.com/janelia-flyem/dvid

4 https://itk.org

5 https://conda.io

6 https://github.com/janelia-flyem/NeuTu

7 https://github.com/janelia-flyem/NeuTu/blob/master/neurolabi/doc/user_manual/neutu/quick_start.pdf

8 https://github.com/janelia-flyem/NeuTu/blob/master/neurolabi/doc/user_manual/neutu/manual.pdf

9 http://neuromorpho.org
